# Feasibility of Collecting and Linking Digital Phenotyping, Clinical, and Genetics Data for Mental Health Research: Pilot Observational Study

**DOI:** 10.2196/71377

**Published:** 2025-06-23

**Authors:** Joanne R Beames, Omar Dabash, Michael J Spoelma, Artur Shvetcov, Wu Yi Zheng, Aimy Slade, Jin Han, Leonard Hoon, Joost Funke Kupper, Richard Parker, Brittany Mitchell, Nicholas G Martin, Jill M Newby, Alexis E Whitton, Helen Christensen

**Affiliations:** 1 Black Dog Institute, University of New South Wales, Sydney, Australia; 2 Department of Neurosciences, Center for Contextual Psychiatry, KU Leuven, Leuven, Belgium; 3 Division of Arts and Sciences and Centre for Global Health Equity, New York University Shanghai, Shanghai, China; 4 Applied Artificial Intelligence Institute, Deakin University, Melbourne, Australia; 5 Brain and Mental Health Program, QIMR Berghofer Institute of Medical Research, Brisbane, Australia; 6 School of Psychology, University of New South Wales, Sydney, Australia; 7 Black Dog Institute, University of New South Wales, Hospital Road, Randwick, 2031, Australia, 61 293828507

**Keywords:** data linkage, precision medicine, experience sampling methodology, daily diary, depression, anxiety, anhedonia, suicidal ideation

## Abstract

**Background:**

Digital phenotyping—the use of digital data to measure and understand behavior and internal states—shows promise for advancing predictive analytics in mental health, particularly when combined with other data sources. However, linking digital phenotyping data with sources of highly sensitive clinical or genetic data remains rare, primarily due to technical, ethical, and procedural challenges. Understanding the feasibility of collecting and linking these data types is a critical first step toward developing novel multimodal datasets.

**Objective:**

The Mobigene Pilot Study examines the feasibility of collecting smartphone-based digital phenotyping and mental health data and linking it to genetic data from an existing cohort of adults with a history of depression (ie, the Australian Genetics of Depression Study). This paper aims to describe (1) rates of study uptake and adherence; (2) levels of adherence and engagement with daily mood assessments; (3) willingness to take part in similar research; and (4) whether feasibility indicators varied according to mental health symptoms.

**Methods:**

Participants aged 18‐30 years with genetic data from the Australian Genetics of Depression Study were invited to participate in a two-week digital phenotyping study. They completed a baseline mental health survey and then downloaded the MindGRID digital phenotyping app. Active data from cognitive, voice, and typing tasks were collected once per day on days 1 and 11. Daily momentary assessments of self-reported mood were collected on days 2‐10 (once per day for 9 days). Passive data (eg, from GPS, accelerometers) were collected throughout the two-week period. A second mental health survey was then completed after two weeks. To measure feasibility, we examined metrics of study uptake (eg, consent) and adherence (eg, proportion of completed momentary assessments), and willingness to participate in similar future research. Pearson correlations and *t* tests explored the relationship between feasibility indicators and mental health symptoms.

**Results:**

Of 174 consenting and eligible participants, 153 (87.9%) completed the baseline mental health survey and 126 (72.4%) provided data enabling linkage of genetic, self-report, and digital data. After removal of duplicates, we found that 100 (57.5%) of these identified as unique participants and 69 (39.7%) provided complete post-study data. A small proportion of participants dropped out prior to completing the baseline survey (21/174, 12.1%) or during app-based data collection (31/174, 17.8%). Participants completed an average of 5.30 (SD 2.76) daily mood assessments. All 69 (100%) participants who completed the post-study surveys expressed willingness to participate in similar studies in the future. There was no significant association between feasibility indicators and current mental health symptoms.

**Conclusions:**

It is feasible to collect and link multimodal datasets involving digital phenotyping, clinical, and genetic data, although there are some methodological and technical challenges. We provide recommendations for future research related to data collection platforms and compliance.

## Introduction

There has been increasing investment in the collection and integration of multimodal datasets to facilitate the development of more precise methods for detecting and predicting complex symptom dynamics in mental health [1,2]. One promising area of inquiry is the integration of smartphone–collected digital phenotyping data with genetic data [3,4].

Digital phenotyping is the process of using digital data to measure and understand behavior and internal states [5,6]. Digital data can be collected passively, for example via accelerometers, or actively, such as through cognitive tasks and the Experience Sampling Method (ESM) [7]. The ESM uses repeated self-report surveys to assess experiences in daily life and has minimal recall biases and high ecological validity [8,9]. Daily diaries are a special form of ESM in which assessments only occur once per day, typically within a prespecified time window [8]. There is evidence that digital phenotyping data can correlate with, classify, and predict mental health problems including depression and anxiety [10-17].

To our knowledge, no previous studies have linked newly collected smartphone-based digital phenotyping data with existing genetic data in mental health research. Creating such a dataset could enhance our understanding of how genetic, behavioural, cognitive, and psychological factors interact in mental health [3,4], offering the potential for better prediction and clinical decision-making [18,19].

### The Mobigene Pilot Study

The purpose of the Mobigene pilot study is to explore the feasibility of collecting digital phenotyping data (ie, from cognitive, voice, and typing tasks; daily diaries; and passive sensors) and self-reported clinical data, from participants in the Australian Genetics of Depression Study (AGDS). The AGDS is an ongoing study examining the contribution of genetic variation to risk of depression in a large cohort of Australian adults [20]. The aims of this paper are to (1) describe rates of study uptake and adherence to the Mobigene study protocol; (2) describe levels of adherence and engagement with daily diary assessments; (3) identify openness to participate in similar research in the future (ie, sustainability); and (4) determine whether feasibility indicators differ according to participants’ current mental health symptoms.

### Transparency and Openness

The postregistered analysis plan [21], deviations, and code are available on the Open Science Framework project page for this study [22].

## Methods

### Design and Procedures

The Mobigene study is an observational pilot study that combines primary and secondary data. Primary data include self-reported current mental health symptoms, active and passive digital phenotyping, and app or phone metadata. Secondary data include existing genetic and historical clinical data collected through the AGDS.

**Figure 1.**
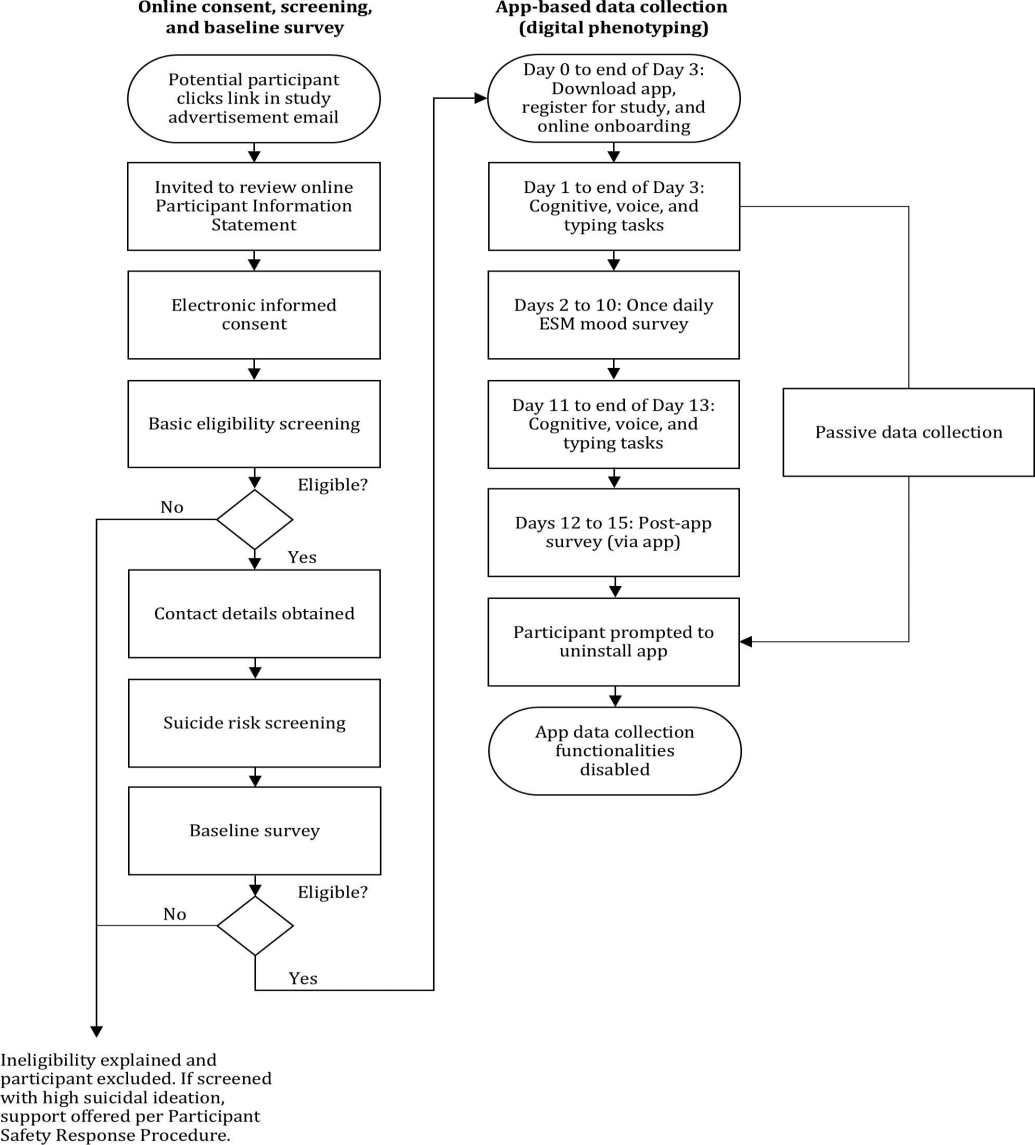
Overview of study procedures including online consent, screening, the baseline survey, and digital phenotyping. ESM: Experience Sampling Methodology.

In total, 1282 participants aged 18‐30 years who had provided genetic data in the AGDS were invited by the AGDS team to participate. Interested participants completed informed consent and eligibility screening. Eligibility criteria included being an Australian resident, owning an iOS smartphone, willingness to participate, and proficiency in English. Eligible participants completed baseline demographic and mental health surveys via Qualtrics and were then invited to download a purpose-built iOS smartphone app for digital phenotyping (MindGRID) on their own smartphones. On days 1 and 11, participants accessed cognitive, voice, and typing tasks in the app. Participants were given 3-days to complete the tasks and each task could only be completed once. From days 2‐10, participants completed a 9-day daily diary period which combined interval- and event-based sampling. In line with best practices [8], for the interval-contingent scheme, participants received one assessment notification from the app per day, sent in the evening at a random time between 7 PM-9 PM. On days 12‐15, depending on the timeliness of task completion, postsurveys assessing mental health and perceptions about the study were readministered via the app. See Figure 1 for study procedures.

Data were collected over two participant cohorts with differing incentive schemes. Participants from cohort 1 could re-enroll in cohort 2 for the opportunity to receive the increased incentive. Duplicate records from cohort 2 were identified and excluded from analyses. The recruitment target was 300 participants and accounted for potential dropout. See Multimedia Appendix 1 for details on eligibility, recruitment, informed consent, and the study protocol (including app-based tasks).

### Ethical Considerations

Ethical approval was obtained from the University of New South Wales, Sydney Human Research Ethics Committee (HC220228) and QIMR Berghofer Human Research Ethics Committee (P3476). Participants provided online informed consent and all study data were deidentified. For compensation, participants were either entered into a draw for 10 Aus $100 (US $65) e-gift cards (cohort 1) or received an Aus $50 (US $33) e-gift card (cohort 2).

### Data Linkage

Three unique identifiers were randomly assigned to each participant: one upon receipt of the study invitation; one during screening; and one upon confirmation of eligibility. These identifiers enabled linkage between baseline and digital phenotyping data for participants from AGDS. See Multimedia Appendix 1 for further details about linkage and privacy.

### Study Uptake and Adherence

Study uptake was defined as the number of eligible participants who consented to participate, the number (%) of participants whose data could be successfully linked, and the number (%) of duplicates. Study adherence was defined as the number (%) of participants that completed baseline and post self-report surveys, as well as the number (%) of participants that dropped out during the study.

### Sample Characteristics

Baseline self-report data included age, education, relationship status, number of children, and current mental health symptoms and treatment. Standardized scales included the Suicide Ideation Attributes Scale [23], Patient Health Questionnaire 9-item [24], Generalized Anxiety Disorder 7-item Questionnaire [25], Snaith-Hamilton Pleasure Scale [26], and Short Health Anxiety Inventory [27]. Total scores and cut-off points for clinical severity were computed (See Table S1 in Multimedia Appendix 1 for internal consistency) [23-31].

### Daily Diary Adherence and Engagement

MindGRID metadata were used to compute adherence to the daily diary protocol. We computed three indices of adherence: the average number (%) of completed evening assessments, the number (%) of participants that did not complete any diaries, and the number (%) of dropouts during the diary period. Engagement was defined as the average number of completed evening and event-based assessments.

### Sustainability

Self-reported openness to take part in a similar future study was assessed postsurvey.

## Results

### Study Uptake and Adherence

**Figure 2.**
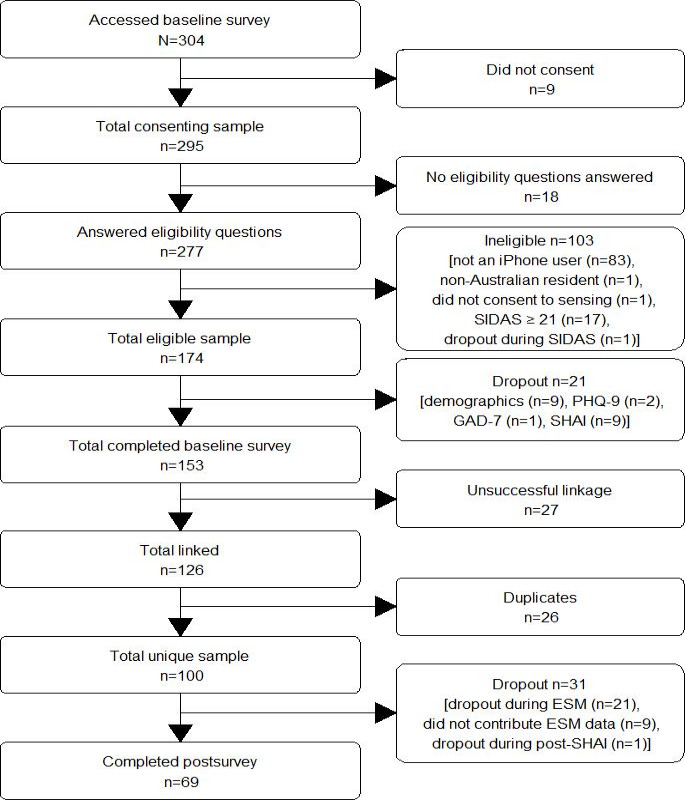
Participant flow diagram illustrating the number of participants at each stage, from initial contact through to study completion. ESM: Experience Sampling Methodology; Generalized Anxiety Disorder 7-item Questionnaire; Patient Health Questionnaire 9-item; Short Health Anxiety Inventory; SIDAS: Suicide Ideation Attributes Scale.

A total of 1,292 participants from the AGDS study were invited to participate. Of the invited participants, 174 eligible participants consented to participate; 21 (12.1%) participants dropped out, leaving 153 (87.9%) who completed the baseline survey. Of these, 126 (72.4%) participants had successfully linked data. Linkage failures were attributed to technical errors: (1) an identifier was not appropriately generated during eligibility; or (2) the eligibility identifier was not recorded due to a bug in the cognitive tasks (ie, if tasks were not completed or timed out by the end of day 3, the identifier was not recorded). After removing 26 duplicates (all from cohort 2), 100 (57.5%) unique participants remained. Thirty-one (17.8%) participants dropped out during the app data collection phase; there were no significant differences in dropout rates between the recruitment cohorts (see Table S2 in the Multimedia Appendix 1). Overall, 69 (39.7%) unique participants with linked data completed postsurveys. The flow diagram is depicted in Figure 2.

### Sample Characteristics

Baseline sample characteristics are reported in Table 1.

**Table 1.** Baseline demographic and mental health characteristics of the total unique sample (n=100) and final postsurvey sample (n=69).

	Total unique sample^a^ (n=100)	Final unique sample (n=69)
Age, mean (SD; range)	27.09 (2.05; 20‐30)	27.13 (2.15; 20‐30)
Education, n (%)		
High school	34 (34.0)	24 (34.8)
Undergraduate	40 (40.0)	27 (39.1)
Postgraduate	26 (26.0)	18 (26.1)
Relationship status, n *(%)*		
Single	39 (39.0)	26 (37.7)
In a relationship	26 (26.0)	19 (27.5)
De facto	18 (18.0)	14 (20.3)
Married	17 (17.0)	10 (14.5)
Number of children, mean (SD*;* range)^b^	0.21 (0.99; 0‐9)	0.13 (0.42; 0‐2)
Mental health symptoms (continuous), mean (SD*;* range)		
Suicidal ideation	5.42 (6.26); 0‐20	4.96 (5.99); 0‐20
Depression	10.99 (5.34); 0‐22	11.07 (5.23); 2‐22
Generalised anxiety	8.95 (5.09); 0‐21	9.32 (5.21); 1‐21
Anhedonia	2.93 (3.15); 0‐13	2.84 (3.24); 0‐13
Health anxiety^c^	20.18 (8.31); 3‐43	20.15 (8.37); 3‐39
Mental health symptoms (≥ clinical cut-off), n (%)^d^		
Suicidal ideation (**≥**1)	66 (66.0)	45 (65.2)
Depression (≥10)	61 (61.0)	42 (60.9)
Generalised anxiety (≥8)	52 (52.0)	36 (52.2)
Anhedonia (≥3)	45 (45.0)	29 (42.0)
Currently taking prescribed medications for mental health, n (%)		
Yes	68 (68.0)	47 (68.1)
No	32 (32.0)	22 (31.9)
Currently receiving psychological therapy for mental health, n (%)		
Yes	50 (50.0)	35 (50.7)
No	50 (50.0)	34 (49.3)

a Total unique sample refers to the total sample after removal of duplicate records.

b n=2 missing.

c n=1 missing.

d There is no established cut-off score for the Short Health Anxiety Inventory [32,33].

### Daily Diary Adherence and Engagement

The average number of evening assessments completed (out of 9) was 5.30 (SD 2.76; range 0‐9; equivalent to 58.9%). The average number of completed evening and event-based assessments was 7.21 (SD 3.79, range 0‐19). Most app-phase dropouts occurred during the diary period (21/31, 67.7%); 9 (29.0%) participants did not complete any diaries. The average diary adherence and engagement was comparable across recruitment cohorts (see Tables S3 and S4 in Multimedia Appendix 1).

### Sustainability

All participants who completed the postsurvey (69/69, 100.0%) reported that they would participate in a similar study in the future.

### Current Mental Health Symptoms and Feasibility Indicators

There were no significant associations between symptoms and dropout (all *t*<1.12; all *P*>.05) or diary adherence and engagement (all *r*<0.19; all *P*>.05) (see Tables S5 and S6 in Multimedia Appendix 1 for statistics).

## Discussion

### Principal Findings

The Mobigene pilot study demonstrated that it was feasible to collect and link new data from an existing cohort that had already participated in extensive data collection procedures. Uptake and adherence to the study protocol were promising; most participants completed at least some daily diaries and there was evidence supporting sustainability. No associations were observed between current mental health symptoms and dropout, daily diary adherence, or engagement.

However, there were methodological and technical challenges during different study phases. Most (n=83) of the ineligible participants did not have an iOS smartphone to support the MindGRID app. Further, technical errors prevented linkage of app data to baseline data for some participants (n=27), and dropout was most common during the digital phenotyping phase (n=31). It is unclear whether dropout was due to issues with daily diaries (eg, timing of assessments), cognitive tasks, or other design-related factors, although it likely reflects the intensive nature of the multimodal data collection protocol. Gathering follow-up data on participants’ subjective experiences of the study, and reasons for noncompletion or dropout is crucial for fully assessing feasibility. Uptake and adherence rates were broadly aligned with findings from other studies using remote monitoring technologies in individuals with a history of depression [34-36]. However, comparisons are challenging given the lack of uptake and adherence standards in intensive digital phenotyping protocols and differences in study design.

### Limitations

First, small sample size limits generalizability of the findings. Second, our estimate of sustainability is likely to be inflated due to self-selection bias, as the 69 participants who completed the postsurvey may represent a particularly motivated subgroup. Third, a large participant pool was required to recruit the final sample.

### Conclusions

Using multimodal data, including integration of existing datasets, is a novel approach to advance mental health prediction and minimize research waste. Our findings indicate that future studies should use a cross-platform data collection apps (eg, both iOS and Android), consolidate data collection via a single mobile platform, and implement adaptive incentives such as gamification to increase compliance [37]. This approach would improve data quality and quantity by broadening eligibility, minimizing technical linkage errors, and increasing participant engagement with data collection procedures.

## Supplementary material

10.2196/71377 Multimedia Appendix 1 Supplementary materials.

## Abbreviations

AGDS: Australian Genetics of Depression Study
ESM: Experience Sampling Methodology
