# Supplementary material for: Feasibility of Collecting and Linking Digital Phenotyping, Clinical, and Genetics Data for Mental Health Research: Pilot Observational Study
Source: JMIR Form Res. 2025 Jun 23;9:e71377. doi: 10.2196/71377 (PMC12207935; doi:10.2196/71377)
Supplement: Multimedia Appendix 1 [file formative-v9-e71377-s001.docx]

# Supplementary Materials

**Eligibility Criteria**

Individuals from the Australian Genetics of Depression Study who had provided genetic data were eligible if they were currently 18-30 years of age; currently residing in Australia and planning to be a resident throughout the study; owned an eligible personal smartphone (iOS); willing to enable passive sensing/routine app data collection for approximately 2-weeks; willing to complete a range of self-report questionnaires and cognitive tasks; and proficient in the English language. Note that those with self-rated suicidal ideation on the Suicide Ideation Attributes Scale (SIDAS) ≥ 21 (“high ideation”) at screening were ineligible. Due to system errors at screening, three participants that had a SIDAS score ≥ 21 were included in the study procedures; we retrospectively classified these participants as satisfying exclusion criteria and removed their data from subsequent analyses.

**Recruitment and Informed Consent**

An email of invitation was sent to 1,282 participants from the eligible AGDS sample frame to participate in the Mobigene study. Those who were interested were prompted to access and read an online version of the participant information statement and, if appropriate, to complete electronic informed consent followed by online self-screening against basic eligibility criteria. This consent form clearly indicated that participants’ existing genetic data would be linked to newly collected clinical and digital phenotyping data. Potentially eligible participants were then asked to provide contact details and to complete screening concerning suicidal ideation.

Data were collected across two cohorts due to lower than anticipated recruitment (note that a reminder email was sent for each cohort). This second cohort had a different incentive scheme to assess whether this would improve feasibility of the study. Incentivisation differed across the two cohorts. In the first cohort, participants who completed the final survey were offered one of 10 AUD$100 e-gift cards through a draw in recognition of their time and contribution to the study. They were also given the opportunity to receive a summary of self-report data they provided throughout the study. In the second cohort, participants who completed the final survey were reimbursed with a $50 e-gift card. Given that incentives were increased in Cohort 2, there were ethical concerns about ensuring participants had the same opportunity to receive the increased reimbursement. Therefore, participants were permitted to sign up again, permitting the occurrence of duplicates in Cohort 2. Duplicates were removed, such that participants were only represented once in the dataset.

**Data Privacy and Linkage**

Identifiable participant information necessary for the administration of the study and/or participant safety follow up, such as contact details and participant follow-up records, were accessible only to named members of the research teams who were involved in these procedures (and who had ethical approval to do so). Identifiable details were stored separately from other study data and protected with encryption.

The linkage process was determined through consultation with QIMR Berghofer Institute of Medical Research to align with their existing data flow. The process involved linking three separate identifiers (one assigned during the AGDS, one assigned during screening, one assigned upon eligibility). All identifiers were de-identified and stored in a different encrypted database from the one that stored identifying information.

Linkage involved the following steps. The AGDS study ID was included in the personalised email of invitation sent to each participant. To facilitate linkage, participants were asked to enter this study ID during Qualtrics screening. When the participant completed screening, a new Qualtrics ID was automatically generated and stored along with the AGDS ID in the screening data file. A list of these IDs was securely sent by the Black Dog Institute team to the Deakin team. When the participant completed download/registration via the study app, including providing a secure one-time password to access the app, a final Participant ID was randomly generated. The Deakin team then securely sent the Black Dog Institute Team a matching file with the Qualtrics ID matched to the Participant ID, thus enabling the Black Dog Institute Team to link the Qualtrics ID and Participant ID with the AGDS ID.

Linkage between data collected in the current study and polygenic risk scores, as well as the phenotypes themselves, will be completed by Black Dog Institute. This process has not yet been completed (only IDs have been linked from the three datasets to demonstrate feasibility). QIMR routinely shares this type of genetic information with collaborative research teams and this possibility is included in their informed consent procedures. Note that shared data will not include individual genomes. As per a data sharing agreement developed by QIMR, genetic data will only be transferred to the Black Dog Institute team and given the sensitive nature, will not be made available to other external researchers. This agreement means that only linked digital phenotyping and survey data collected in the current study can be accessed for future research purposes. Data access can occur via a data access request, which will be reviewed via the study publications committee. These procedures were indicated in the study participant information statement and consent form.

**Study Tasks**

Cognitive, typing, and voice tasks were included in the current study because they have the capability to provide mechanistic insight into the way emotional regulation and cognitive function affect psychological outcomes. These tasks were based on those included in a longitudinal cohort study of 6388 adolescents in Australia (1), which were collected alongside other digital phenotyping data via a purpose-built app (Future Proofing). Given that specific details about these tasks have been reported elsewhere (1-5), we provide a summary below. The phone-based delivery of these tasks has not been extensively validated in adult samples with a history of depression.

**Cognitive Tasks**

1. Card Sorting Task

This task assesses shifting ability via ability to deduce logical rules in a card game-like format with/without affective images. Non-affective images included shapes; affective images included faces. In this task, participants were dealt a card and had to sort it to one of four decks according to three possible sorting rules: card colour, number of items, and shape (cognitive control condition) or emotional expression (affective control condition). Performance is typically operationalised as accuracy (i.e., proportion of random errors) and reaction time on colour and number trials.


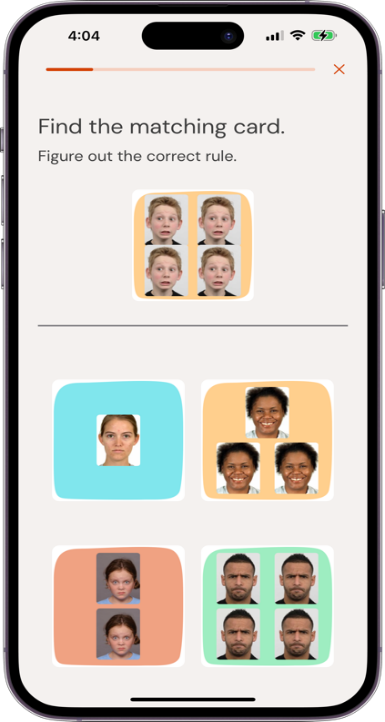

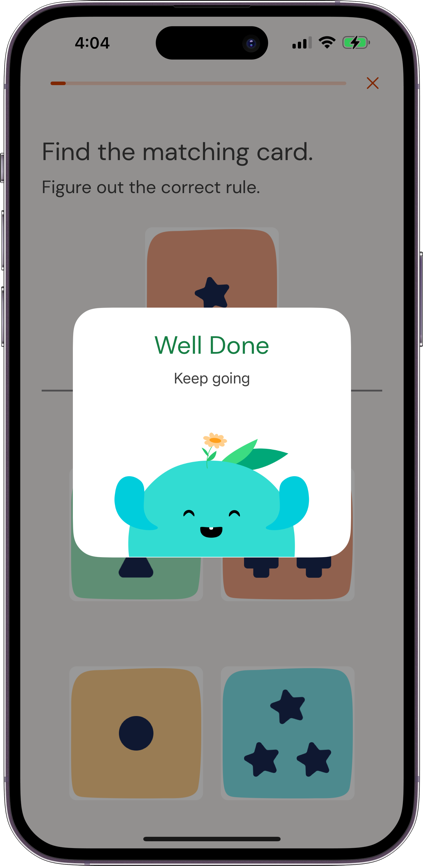

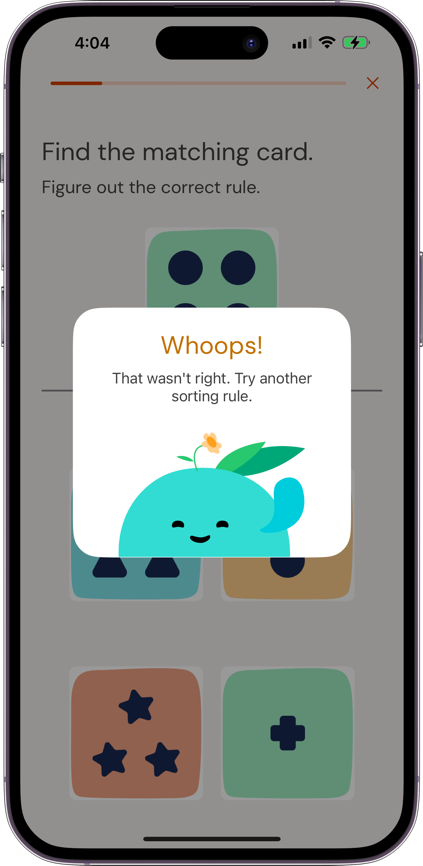


2. Backward Digit Span Task

This task assesses working memory via the ability to recall numbers of increasing length with/without affective images. Non-affective images include furniture; affective images include people/animals. In this task, participants were presented with digits in serial order to be recalled in reverse order. Digits were either superimposed over neutral images (cognitive control condition) or over negative images (i.e., affective control condition). Performance is typically operationalised as the maximum span level achieved.


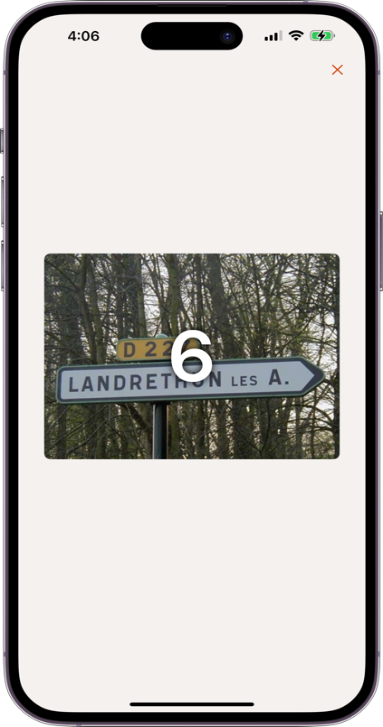

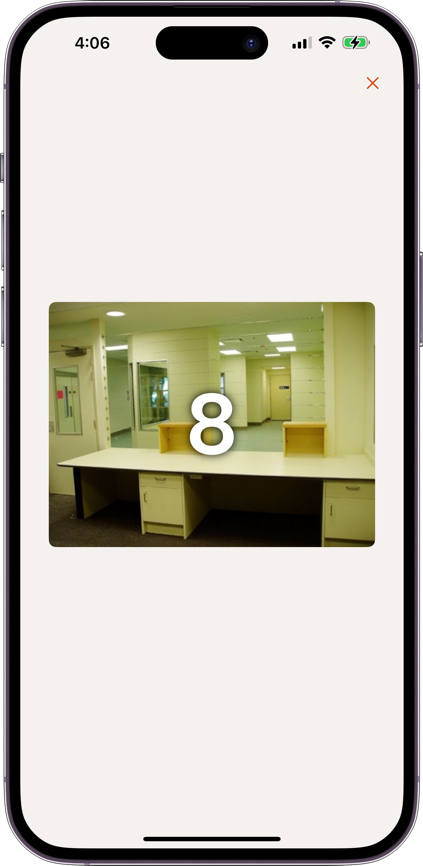

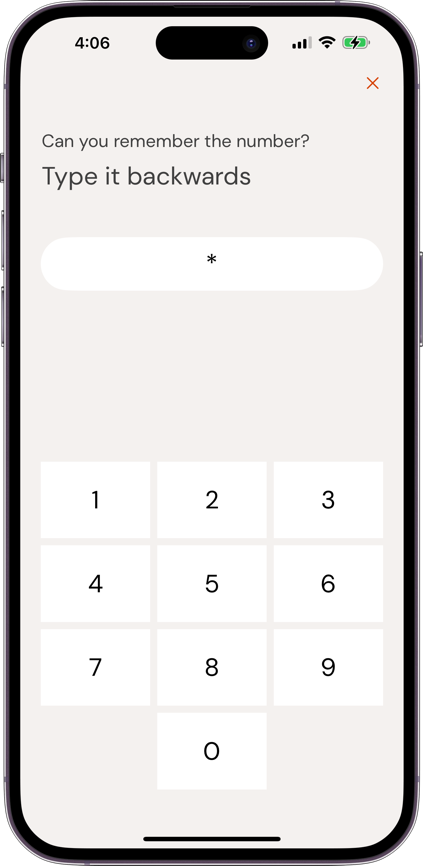


**Typing Tasks**

Two short typing tasks asked participants to transcribe a short sample of provided text (prose task) and compose a response to a question (composition task). In the prose task, participants had 30 seconds to copy (type) as much text as possible. In the composition task, they were asked to type between 200-500 characters on a randomly given topic without any time limit. The typing task did not include identifiable information.


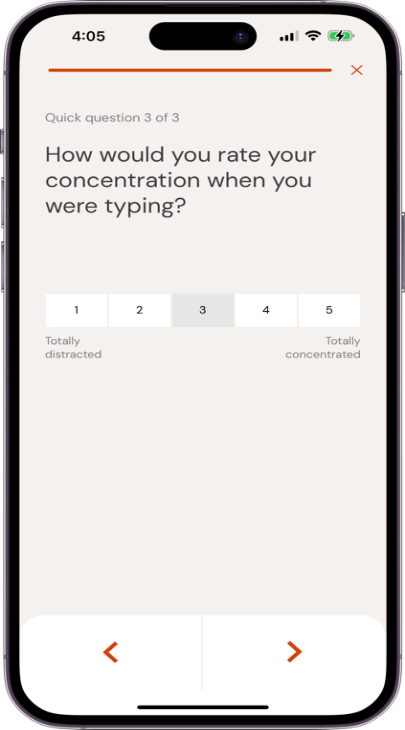

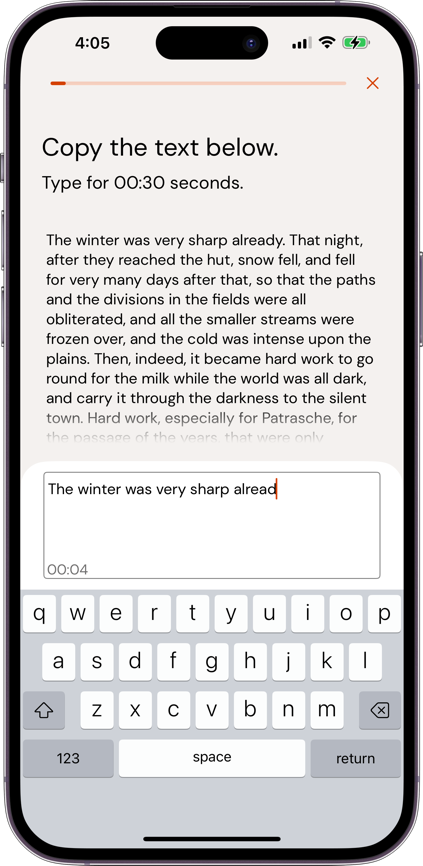


**Voice Tasks**

Three short voice tasks asked participants to provide brief samples of stereotyped speech. They include making sounds (e.g., repeated vowel sounds), reading text out loud, and recounting a story out loud. Samples did not incorporate identifying elements.


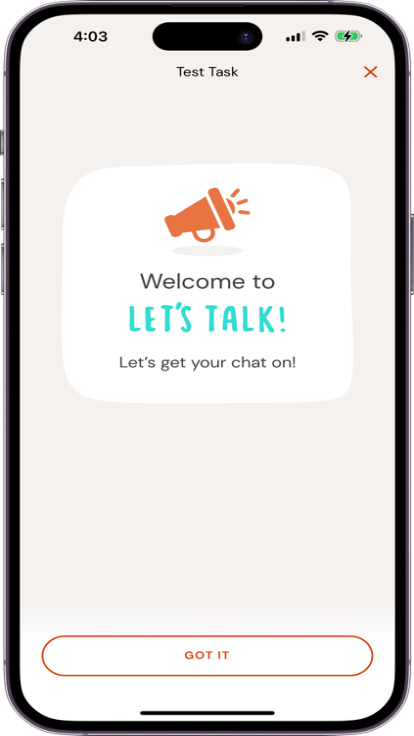

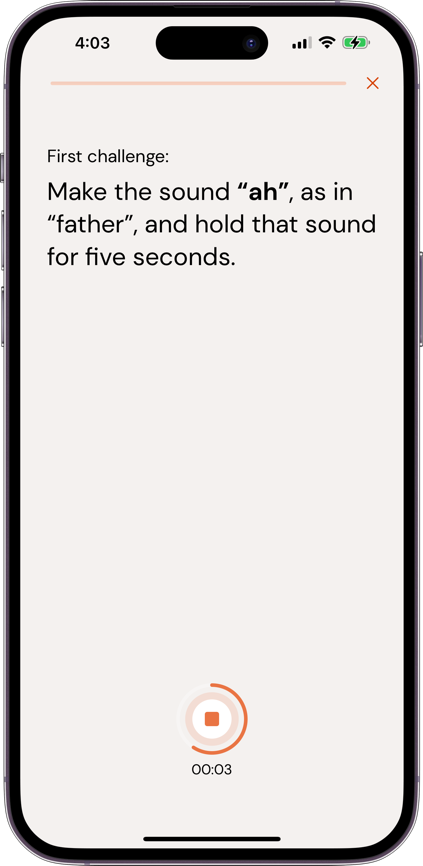

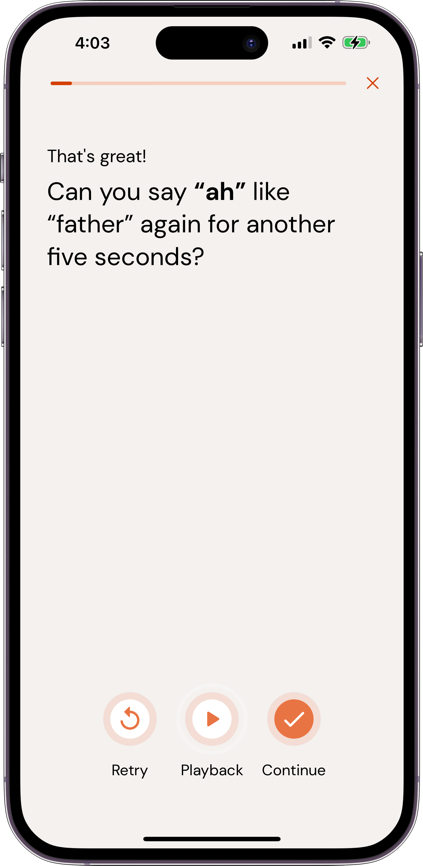


**References**

1. Werner-Seidler A, Huckvale K, Larsen ME, Calear AL, Maston K, Johnston L, Torok M, O’Dea B, Batterham PJ, Schweizer S, Skinner SR. A trial protocol for the effectiveness of digital interventions for preventing depression in adolescents: The Future Proofing Study. Trials. 2020 Dec;21:1-21.
2. Braund TA, O’Dea B, Bal D, Maston K, Larsen M, Werner-Seidler A, Tillman G, Christensen H. Associations between smartphone keystroke metadata and mental health symptoms in adolescents: Findings from the future proofing study. JMIR mental health. 2023 May 15;10:e44986
3. Minihan S, Kumle L, Maston K, Bal D, Werner‐Seidler A, Christensen H, Schweizer S. The relationship between cognitive and affective control and adolescent mental health. JCPP advances. 2024 Mar;4(1):e12204.
4. Schweizer S, Leung JT, Kievit R, Speekenbrink M, Trender W, Hampshire A, Blakemore SJ. Protocol for an app-based affective control training for adolescents: proof-of-principle double-blind randomized controlled trial. Wellcome Open Research. 2019 Oct 2;4:91.
5. Schweizer S, Parker J, Leung JT, Griffin C, Blakemore SJ. Age-related differences in affective control and its association with mental health difficulties. Development and psychopathology. 2020 Feb;32(1):329-41.

**Mind GRID App Study Protocol**

**Note:** Similar to the Vibe Up app, the Mind GRID app uses a game-like mechanism to promote adherence. The mechanism is based on the evolution of a virtual character “Sprout” from infancy (an eggshell) to adulthood throughout the study period. See the Vibe Up study protocol for additional details (Huckvale et al, 2023; doi: <https://doi.org/10.1136/bmjopen-2022-066249>). EMA refers to Ecological Momentary Assessment (EMA), otherwise known as Experience Sampling Methodology (ESM).

**DOWNLOAD PHASE** *(Days 0 - end of Day 3)*

1. Users receive an SMS on Day 0 at x time.
2. User clicks the link to download the app from Testflight.
3. Refer to Vibe Up protocol for the SMS and Email reminder behaviour to get participants to download the app.

**REGISTRATION & ONBOARDING PHASE** *(Days 0 - end of Day 3)*

1. Users enter their mobile number and register with one-time password (OTP).
2. Users undergo onboarding with permissions.
3. App does the registration process.
4. User steps through app tour.

**STUDY PHASE 1:** 4 COGNITIVE, VOICE, TYPING TASKS *(Days 1 - end of Day 3)*

1. Users will see the Welcome to Phase 1 screen.
2. **Phase 1** is to complete the 4 tasks.
3. Users can attempt each Task only once.
4. The event log will need to record the incomplete attempt and the app will need to persist data was gleaned/captured before the user abandoned or killed the task.
5. Users get a daily reminder notification to do them, 1000 hrs (aka 10am) in the first 3 days of the study.
   1. ****If users complete the tasks:**** they are deemed to have progressed to Phase 2. Completion of all 4 will show the bottom panel of Welcome to Phase 2, similar to Vibe Up.
      1. The catch here is, the initial 3 day window to complete the tasks will show Sprout day 1 to day 3, which depicts the eggshell. Once they complete Phase 1 and progress to Phase 2, Sprout starts from Sprout 4 (without eggshell).
   2. ****Else:**** Users do not get kicked out of the study if they fail to complete the tasks, they just progress to Phase 2 once the 3 days have elapsed.
      Users will see an Out of Time screen explaining they have run out of time to complete all tasks.
6. The tasks will expire at the end of Day 3 if the user has not completed / attempted the tasks.
7. **Phase 1 ends** if:
   1. User has completed / attempted all 4 tasks, or
   2. Study has transitioned into Day 4 (midnight). If the study has transitioned into Day 4 without the user having completed the tasks, show the “You ran out of time” bottom sheet. Users will dismiss this sheet to see the Welcome to Phase 2 screen.
8. Users will see the Welcome to Phase 2 screen to indicate the transition.

**STUDY PHASE 2:** EMA AND FEED THE SPROUT *(Days 2 - 10)*

1. This Phase can start on Day 2 or Day 3 (if the user is diligent), or arbitrarily start on Day 4.
2. This Phase arbitrarily ends at the end of Day 10.
3. Phase 2 is the start of **daily** Feed Sprout (morning between 0800 - 1000 to trigger the start of the 1 hour window to complete this task) and EMA (evening between 1900 - 2100 to trigger the start of the 1 hour window to complete this task) tasks.
4. In the mornings between 0800 - 1000 "randomly", users receive a notification that a task is available.
   1. If the task has not been completed, we send another push notification 30 mins later.
5. The feed Sprout looks like a regular activity on the ‘Today's Tasks’ tray.
   1. Clicking it will play a foreground animation that, for example, shows a fruit dropping from the top of the screen, bouncing on the floor, and then a comic style om nom nom bubble appears obscuring the Sprout. The Today's Task will have some manner of 'loading' animation while this animation plays. Once the comic bubble fades out, we transition Sprout to the next day Sprout, and the Today's Tasks show the 'All caught up' bottom pane.
   2. If the 1 hour expires for the feed Sprout task, it disappears and it shows the 'No pending tasks' bottom pane (or the EMA survey); Sprout remains as the current Sprout until midnight when we transition Sprout to the next day Sprout.
6. In the evenings between 1900 to 2100 randomly, we fire the EMA notification, followed by another push 30 mins later if they have not completed it. Behaviour for EMA will largely be the same as Vibe Up (see protocol).
7. Users will see the Welcome to Phase 3 screen to indicate the transition.

**STUDY PHASE 3:** 4 COGNITIVE, VOICE, TYPING TASKS *(Days 11 - 13)*

1. **Phase 3** starts on Day 11 of the study, where the 4 cognitive tasks show up again.
2. **No more EMA and feed Sprout tasks.** The 4 tasks are available for 72 hours, with daily push notifications at 1000 hrs to inform the user they have tasks to do.
3. Users can attempt each task only once.
4. The event log will need to record the incomplete attempt and the app will need to persist data was gleaned/captured before the user abandoned or killed the task.
5. Users get a daily reminder notification to do them, 1000 hrs (aka 10am) in the first 3 days of the study.
   1. ****If users complete the tasks:**** they are deemed to have progressed to Phase 4. Completion of all 4 will show the bottom panel of Welcome to Phase 4, similar to Vibe Up.
   2. ****Else:**** Users do not get kicked out of the study if they fail to complete the tasks, they just progress to Phase 4 once the 3 days have elapsed.
      Users will see an Out of Time screen explaining they have run out of time to complete all tasks.
6. The tasks will expire at the end of Day 13 if the user has not completed / attempted the tasks.
7. **Phase 3 ends** if:
   1. User has completed / attempted all 4 cognitive tasks, or
   2. Study has transitioned into Day 14 (midnight). If the study has transitioned into Day 14 without the user having completed the tasks, show the “You ran out of time” bottom sheet. Users will dismiss this sheet to see the Welcome to Phase 4 screen.
8. Users will see the Welcome to Phase 4 screen to indicate the transition.

**STUDY PHASE 4:** 1 TASK POST-APP SURVEY *(Days 12 - 15)*

1. **Phase 4** will only have 1 task, the Wellbeing Survey, which is a Post-App Survey with relatively simple survey logic.
2. This phase has a similar reminder schedule to Vibe Up Post bundle, with push, SMS and email reminders to complete (see protocol).
   1. Once done, we show the completion type bottom sheet similar to Vibe Up
   2. Otherwise, if they fail to complete the Post-App Survey within the 72 hours, we will show the ran out of time bottomsheet, similar to Vibe Up.
3. All Passive Sensing should cease if:
   1. The user has completed the Wellbeing Survey, or
   2. Day 15 of the study has elapsed, whichever happens earlier.

**Internal Consistency Estimates**

**Table S1.** Cronbach’s alpha for standardised mental health surveys (*N*=100).

| Mental health symptoms | α |
| --- | --- |
| Suicidal ideation (SIDAS) | .84 |
| Depression (PHQ-9) | .85 |
| Generalised anxiety (GAD-7) | .89 |
| Anhedonia (SHAPS) | .83 |
| Health anxiety (SHAI) | .89 |

**Study Uptake and Adherence Across Cohort 1 and Cohort 2**

**Table S2.** Dropout comparison across recruitment cohorts (*N=*100).

| Recruitment cohort | Not dropout  *N* (%) | Dropout  *N* (%) |
| --- | --- | --- |
| Cohort 1 | 37 (69.8) | 16 (30.2) |
| Cohort 2 | 32 (68.1) | 15 (31.9) |

**Daily Diary Adherence and Engagement Across Cohort 1 and Cohort 2**

**Table S3.** Diary average adherence across recruitment cohorts (*N=*100).

| Recruitment cohort | *M* (*SD*) | Range |
| --- | --- | --- |
| Cohort 1 | 4.89 (2.88) | 0–9 |
| Cohort 2 | 5.77 (2.56) | 0–9 |

**Table S4.** Diary average engagement across recruitment cohorts (*N=*100).

| Recruitment cohort | *M* (*SD*) | Range |
| --- | --- | --- |
| Cohort 1 | 6.94 (4.45) | 0–19 |
| Cohort 2 | 7.51 (2.90) | 0–13 |

**Relationships between Current Mental Health Symptoms and Feasibility Indicators**

**Table S5.** T-tests assessing differences in mental health by dropout status (*N=*100).

| Indicator | Suicidal ideation  *t(df)*, *p* | Depression  *t(df)*, *p* | Generalised anxiety  *t(df)*, *p* | Anhedonia  *t(df)*, *p* | Health anxiety^a^  *t(df)*, *p* |
| --- | --- | --- | --- | --- | --- |
| Dropout | 1.05(51.7),  .30 | -0.22(53.9), .82 | -1.12(62.6), .27 | 0.44(62.4),  .67 | 0.06(58.52),  .95 |

^a^*n*=1 missing.

**Table S6.** Correlations between mental health and feasibility indicators (*N=*100).

| Indicator | Suicidal ideation  *r*, *p* | Depression  *r*, *p* | Generalised anxiety  *r*, *p* | Anhedonia  *r, p* | Health anxiety^a^  *r*, *p* |
| --- | --- | --- | --- | --- | --- |
| Diary Adherence | -0.08, .42 | 0.03, .74 | 0.10, .32 | 0.007, .94 | 0.02, .86 |
| Diary Engagement | -0.02, .83 | 0.13, .21 | 0.19, .06 | 0.05, 0.59 | 0.02, .84 |

^a^*n*=1 missing.
